# Comparative genomic analysis of paired clinical isolates from a patient with recurrent melioidosis reveals a low within-host mutation rate

**DOI:** 10.1099/jmm.0.002003

**Published:** 2025-04-15

**Authors:** Sruthi Raj, Sreeram Chandra Murthy Peela, Hithesh Kumar, Sudha Ramaiah, Sujatha Sistla

**Affiliations:** 1 Department of Microbiology, Jawaharlal Institute of Postgraduate Medical Education and Research, Puducherry, India; 2 Department of Biosciences, School of Biosciences and Technology, Vellore Institute of Technology, Vellore, India

**Keywords:** *Burkholderia pseudomallei*, genome sequencing, melioidosis, relapse

## Abstract

**Introduction.** Relapse of melioidosis is not uncommon and can occur due to shorter oral antibiotic therapy in the first episode. In such isolates, low mutation rates were identified amongst paired clinical isolates during relapse, but large-scale structural variants were also common.

**Hypothesis.** Using pair-wise comparison, a low number of mutations, especially amongst the virulence and antibiotic resistance genes, may be present amongst the paired isolates obtained during the study period.

**Aim.** A pair of clinical isolates obtained from a patient with recurrent melioidosis during the study period (January 2018 to June 2021) was analysed for identifying the genomic relatedness and DNA changes that may have caused the relapse.

**Methodology.** Using paired-end Illumina sequencing, following appropriate data quality checks, the genomes were assembled using Shovill pipeline, whilst the variants were called using Snippy. Structural variants were detected using TIDDIT, and functional associations were identified using the STRING database searches.

**Results.** One of the isolates (from the second episode) had a highly fragmented genome, but very few structural variants and SNPs were identified. Both the isolates had similar virulence and antibiotic resistance genes; however, owing to the few structural changes, a slightly lower number of virulence genes were observed. Together, they shared 99.8% of the proteomes, and most variants identified spanned either hypothetical proteins or un-annotated regions.

**Conclusions.** Based on comprehensive genome analysis the two strains were genetically similar, with a few structural variants, implying the second episode to be a relapse rather than a re-infection. There was no difference in the antibiotic resistance or virulence genes that may have explained the relapse.

## Data Summary

All the raw sequencing data (FASTQ) files were submitted in NCBI SRA in the Bioproject PRJNA781752 with accession IDs SRR16979226-7. The intermediate data files (BAM files, etc.) are available at https://figshare.com/projects/Burkholderia_pseudomallei_Clinical_pair/189708.

## Introduction

*Burkholderia pseudomallei* (*B. pseudomallei*) is a highly recombinogenic bacterium found in the environment. Whole-genome sequencing (WGS) helps to trace the origin of *B. pseudomallei* strains and understand how the pathogen spreads and persists in the environment, which is essential to protect both humans and animals from the potentially deadly infectious disease, melioidosis. The genome of *B. pseudomallei* is more complex compared to most bacterial genomes [1]. Moreover, its large size (7.25 Mb) places it in the largest 5% sequenced microbial genome category [2,3]. Several genomic islands are identified in the genome that are acquired through lateral gene transfer adding to the genome plasticity of *B. pseudomallei* [2,3].

Recurrent melioidosis is not an uncommon phenomenon and has been reported from different parts of the world. Using genotyping for comparing the isolates from different episodes, the sequelae of infection can be established as recurrent melioidosis (if isolates are genetically related) or re-infection with a different strain. The classification of recurrent melioidosis is important for assessing treatment failure and identifying epidemiological/genetic factors underlying recurrence/reinfection. In an earlier report from our group, we have identified an episode of melioidosis relapse in a diabetic patient and a farmer by profession where the initial episode was recorded in December 2019 [4]. The patient underwent complete specific therapy but had similar symptoms nine months later (in September 2020), the clinical presentation being multiple leg abscesses and septic arthritis. The patient succumbed to the disease as no hospitalization was provided due to the COVID-19 pandemic at that time. In both episodes, *B. pseudomallei* was detected by culture in pus specimens.

Genome sequencing is emerging as a common trend to study outbreaks and genetic relations amongst the strains. Some of the methods to infer strain similarity are core genome MLST, genome alignment and identification of clusters based on genomes. Whilst these are computationally intensive, simple algorithms to identify sequence types and multi-locus profiles are applied to genome data and are comparatively faster. Previously, a few studies analysed the genomic differences from paired isolates obtained during recurrent melioidosis infections [5,6]. Based on the comparative genomics methods, a mutation rate of 1–3.5 SNPs per year was observed amongst the isolates [6]. However, large-scale genomic deletions, especially within chromosome no. 2, were identified [6]. Therefore, it is important to identify the changes in the genomic composition of the strains and identify the possible causes of recurrent melioidosis by focussing on the distribution of antibiotic resistance and virulence genes.

The present study was thus undertaken to identify the genetic relatedness of the isolates across the two episodes and identify the probable emergence of antimicrobial resistance and genomic alterations amongst the isolates obtained during the two episodes.

## Methods

### Bacterial strains

A pair of clinical isolates from a 45-year-old diabetic farmer with multiple leg abscesses and septic arthritis with recurrent melioidosis was collected and analysed using short-read genome sequencing in the present study [4]. After diagnosis in the first episode, the patient received meropenem for 14 days in the intensive phase and cotrimoxazole in the continuation phase with a duration of 90 days [4]. The relapse occurred after 9 months, and both isolates were susceptible to meropenem, imipenem, ceftazidime, doxycycline, amoxicillin/clavulanic acid and trimethoprim-sulphamethoxazole (cotrimoxazole) by the Kirby–Bauer disc diffusion method.

### DNA purification

DNA was extracted from the isolates 28_Ex_14915 (episode 1, hereafter referred to as EX14915) and 28_Ex_7035 (episode 2, hereafter referred to as EX7035) using QIAamp DNA Blood Mini kit (Qiagen, Hilden, Germany) as per the manufacturer’s instructions. The purified DNA samples were checked for their quality using spectrophotometry (Nanodrop, Thermo-Fisher Scientific, Waltham, USA).

### Whole-genome sequencing

The libraries were prepared using the Illumina TruSeq Nano DNA Library Prep Kit. Two hundred nanograms of gDNA were fragmented by Covaris M220 and were subjected to end-repair using End Repair Mix. AMPure XP beads were used to size-select the adapter-ligated products, which were subjected to PCR using the index primer. The quantity and quality of the libraries were analysed on the 4200 TapeStation system using D1000 Screen tape as per the manufacturer’s instructions. The cluster generation and sequencing of the paired-end Illumina libraries were performed with the NextSeq500 platform using 2×150 bp chemistry targeting 100X coverage. Both samples were processed simultaneously to avoid any technical differences. The basecall files were converted to FASTQ formatted files using the bcl2fastq program. The raw FASTQ files were deposited into the NCBI SRA database under the BioProject PRJNA781752. Default parameters were used for all analyses unless specified.

### Genome assembly and analysis

The raw FASTQ files were assessed for quality using FastQC v0.11.9 (https://www.bioinformatics.babraham.ac.uk/projects/fastqc/). Using the Shovill v1.1.0 pipeline (https://github.com/tseemann/shovill) for automatic read trimming and assembly, the assembled genomes were annotated with Prokka v1.14.6 by selecting the compliance with GenBank [7]. The assembled genomes are used to predict multi-locus sequence types (STs) by the PubMLST web server [8]. Virulence and antibiotic resistance genes were predicted using VFDB [9] and NCBI AMRFinder Plus [10] databases respectively with Abricate v1.0.1 (https://github.com/tseemann/abricate).

From the NCBI RefSeq database, complete genomes of *B. pseudomallei* (Bps) were downloaded (accessed on 21 December 2023) (Supplementary Material 1). The genome assemblies in the present study were compared with the complete genomes to identify the Mash distance of both isolates using Mash v2.0 [11]. The Mash distance is an estimate of the number of mutations (‘differences’) between two genomes. The MSHR7901 genome (belonging to ST735) was selected as a reference for comparative visualization with BRIG v0.95 [12].

From the annotated proteomes, the orthologous protein clusters were identified using OrthoVenn3 using the OrthoMCL algorithm with an E-value 1E-5 and an inflation value of 1.5 [13]. For visualization of protein cluster distribution, ClusterVenn available in the OrthoVenn web server was used. The protein clusters that were unique in either genome were searched against the STRING v12.0 database (https://string-db.org/) for *Burkholderia*, filtering on high-confidence scores (>700) and viewing up to 20 interactors (in shell 2) with the input proteins. Subsequent GO enrichment results from the STRINGdb search were used to identify the gene/cluster functions after applying a false discovery threshold of 0.05.

The SNPs in the relapse isolate were compared by mapping the reads and variant calling with Snippy v4.6.0 (https://github.com/tseemann/snippy) on the isolate from the initial episode (EX14915). The structural variants (SVs) were subsequently identified using the BAM file with TIDDIT v3.6.1 pipeline using default parameters but correcting for ploidy (-n 1) and forcing the ploidy over the entire genome [14]. The coverage function in TIDDIT v3.6.1 was used to identify read coverages across the reference (EX14915) genome.

## Results

### Read quality control and assembly characteristics

**Fig. 1.**
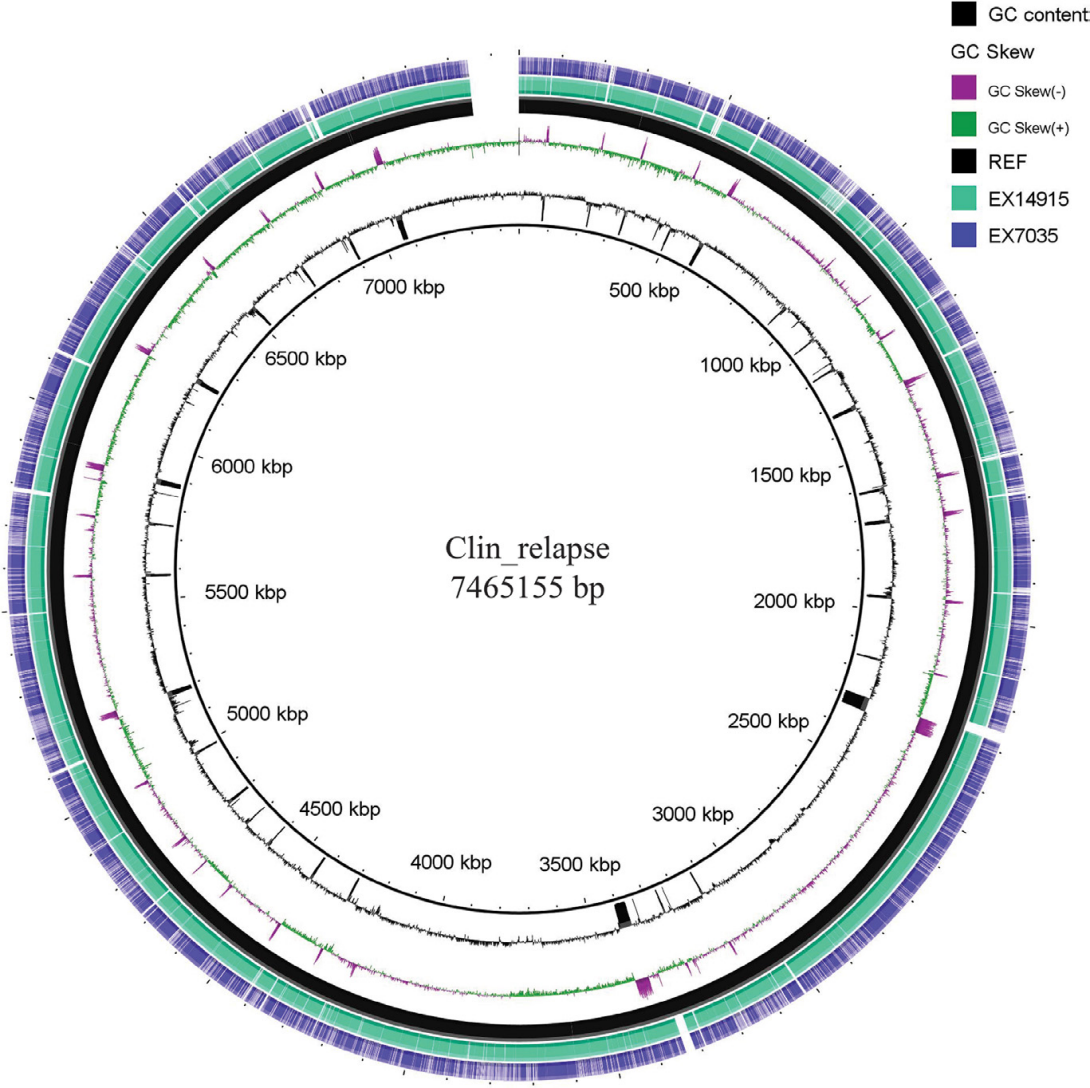
Comparative analysis of two isolates with the reference MSHR7901 genome (black). As observed, the genome EX7035 is highly fragmented (regions of low similarity and breaks) when compared with EX14915. Image created using BRIG v0.95 with max and min identity set at 100 and 70, respectively.

**Fig. 2.**
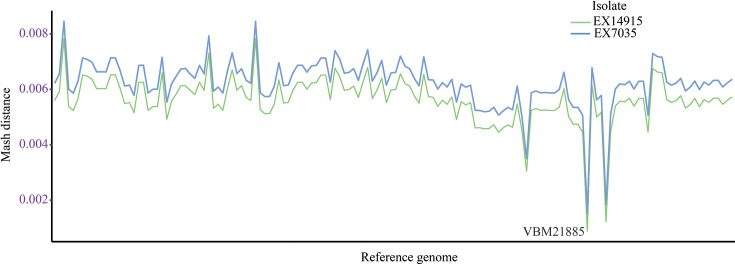
Mash distance of the isolates with respect to the complete genomes available in the RefSeq database. The X-axis represents various genomes and is omitted for image clarity [refer to File S1, for raw data (available in the online version of this article)].

Raw sequence reads from both isolates were analysed for errors in sequencing, and analysis with FastQC revealed that all the reads were of high quality (Phred quality ≥20 in >92% of the bases), with no over-represented sequences or adapter contamination (Table S1). Subsequently, both genome assemblies were constructed using the Shovill pipeline. Initial analyses identified a highly fragmented genome for EX7035 (Table 1, Fig. 1), with low N50 and high number of contigs. Comparing with RefSeq genomes, the VBM21885 genome (RefSeq accession: GCA_017378195.1) was identified as having the least Mash distance of 0.000780658 and 0.00139189 for EX14915 and EX7035, respectively (Fig. 2) (Supplementary Material 1). Both isolates belonged to ST734 with no observed difference in the allelic profiles and were of similar LPS-A and Bim-A_BP_ genotypes.

**Table 1.** Characteristics of genome assembly and annotation of two isolates

Feature	EX14915	EX7035
Episode	First	Second
SRA ID	SRR16979226	SRR16979227
Contigs	451	1,684
Total genome length	7,160,596	6,979,157
GC	68.09	67.9
N50	34,519	7,360
L50	65	246
Genes	6,061	6,080
ncRNA	51	49
tRNA	79	77
rRNA	2	2
Antibiotic resistance genes (NCBI)	2 (blaOXA-57, PenI)	2 (blaOXA-57, PenI)
Virulence genes (VFDB)	138	115
N Prot clusters	5,462	5,457
N singletons	422	457

N50 and L50 are usual genome assembly metrics that define the median contig length and number of contigs in the assembly that have atleast this size respectively.

GC, Percent GC.

### Gene differences

**Fig. 3.**
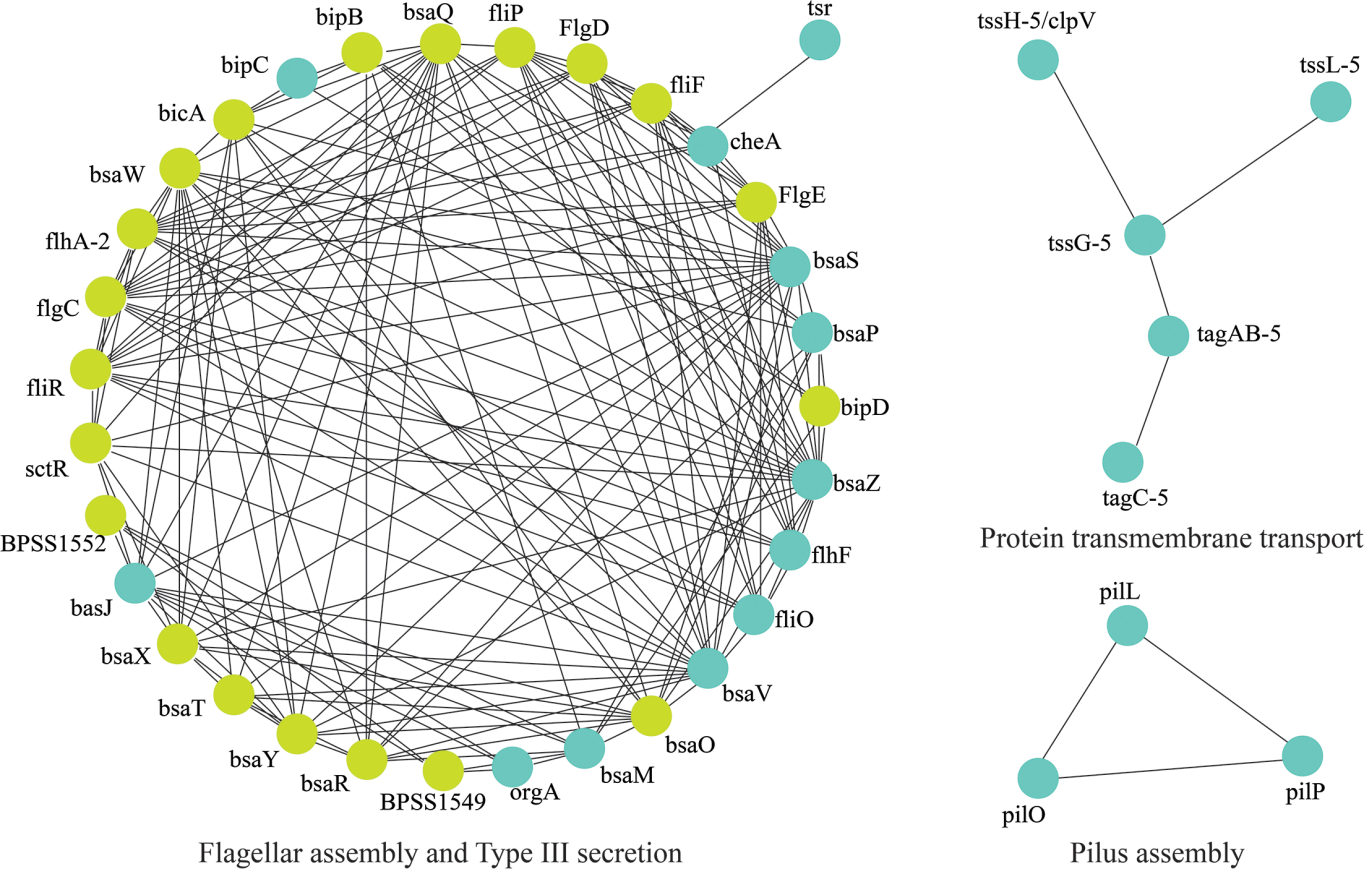
Virulence genes identified exclusively in EX14915. Proteins that are exclusive for the isolate are colored cyan while those from the STRING database are in light green. The clusters are labelled according to their biological function: type III secretion system (GO: 0030254), flagellar assembly (GO: 0044781), pilus assembly (CL: 4673), and protein transmembrane transport (CL: 10280).

**Fig. 4.**
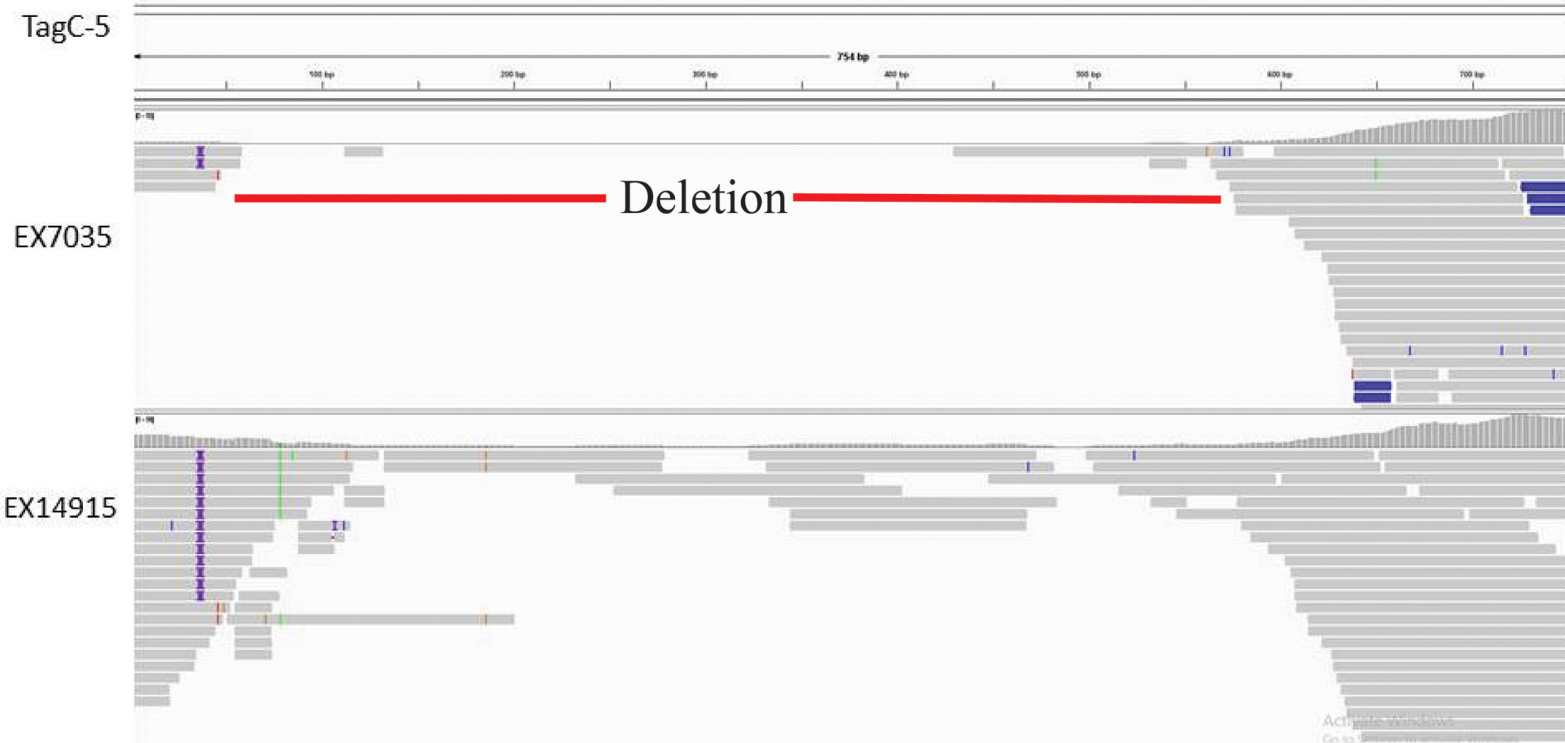
Comparison of read coverages of the tagC-5 gene (as an example) in EX7035 (above) and EX14915 (below). No coverage was observed for the region spanning 50–550 bp in EX7035, with a few reads soft-clipped (indicated in blue) towards the end of the gene. The gene sequences were obtained from VFDB, reads were mapped using Snippy and an image was generated using IGV v2.15.1.

After annotation using Prokka, the isolate from the first episode had a lower number of genes but a higher number of non-coding RNAs (Table 1). The genes contributing to antibiotic resistance were similar in the isolate from the second episode when compared with the initial isolate, whilst the number of virulence genes was reduced (Table 1). Amongst the 23 virulence genes exclusively detected in EX14915, the STRINGdb searches revealed that five genes (bsaS, bsaZ, flgA, flhF and fliO) were enriched with GO: 0044781 (bacterial-type flagellum organization). These five genes were part of a flagellar assembly network as visualized via the STRINGdb protein interaction network (Fig. 3) . Similarly, amongst the other four genes, three genes (bsaV, cheA and tsr) were enriched with GO: 0006935 (chemotaxis) (Fig. 3). The isolate EX7035 had an additional protein CheA of *Yersinia enterocolitica* that is involved in chemotaxis. To identify the reasons for the absence of virulence factors, the reads were mapped to the virulence genes unique to EX14915 using Snippy. Most of these genes had insufficient read coverages in EX7035, due to which these genes were deemed absent in this isolate (Fig. 4 shown for tagC-5 gene).

### Protein clusters

**Fig. 5.**
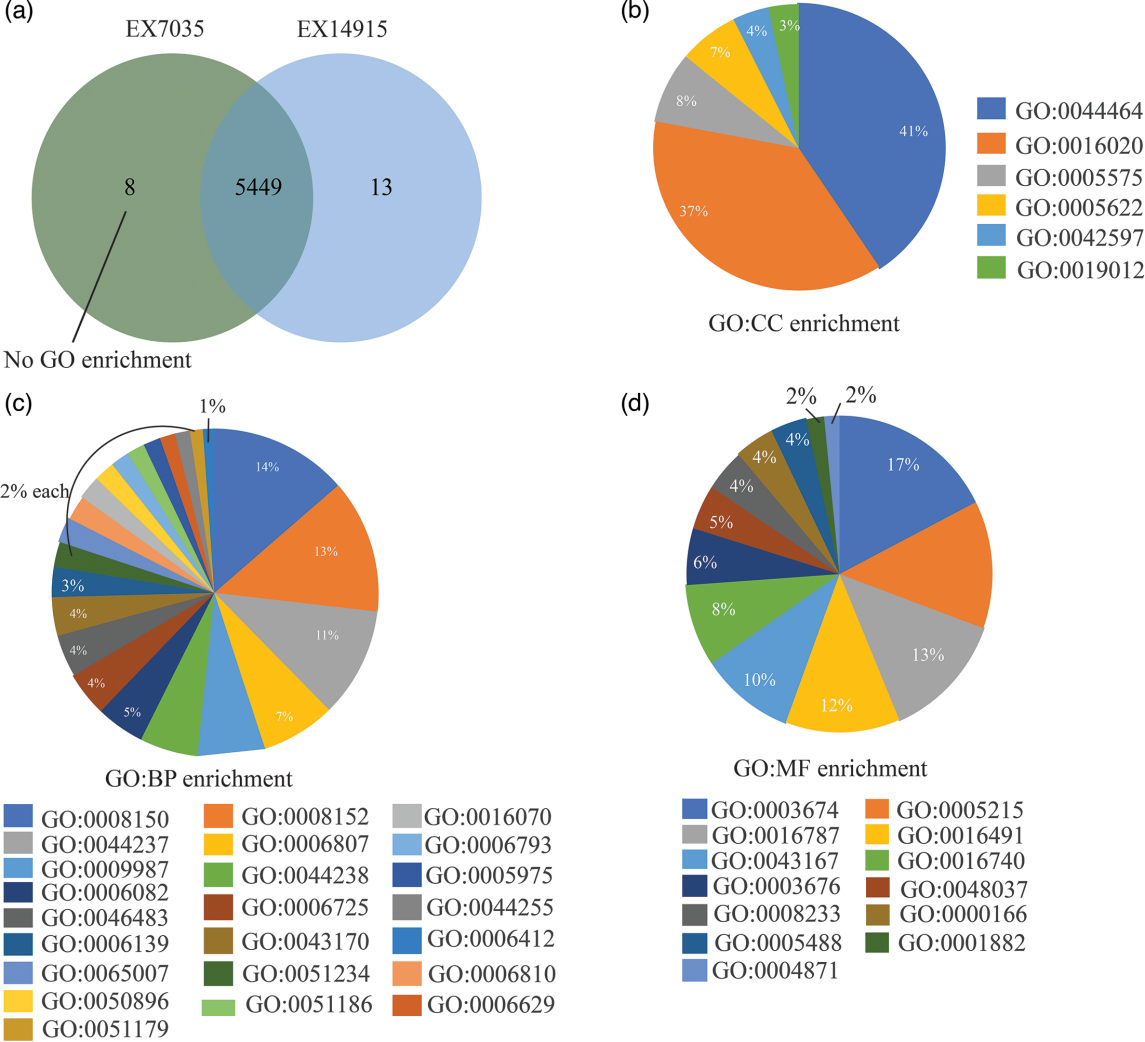
(a) Common and unique clusters amongst the two isolates. (**b–d) **The percentage of genes amongst the 5,449 clusters with each GO term. The figure is obtained using OrthoVenn3.

**Fig. 6.**
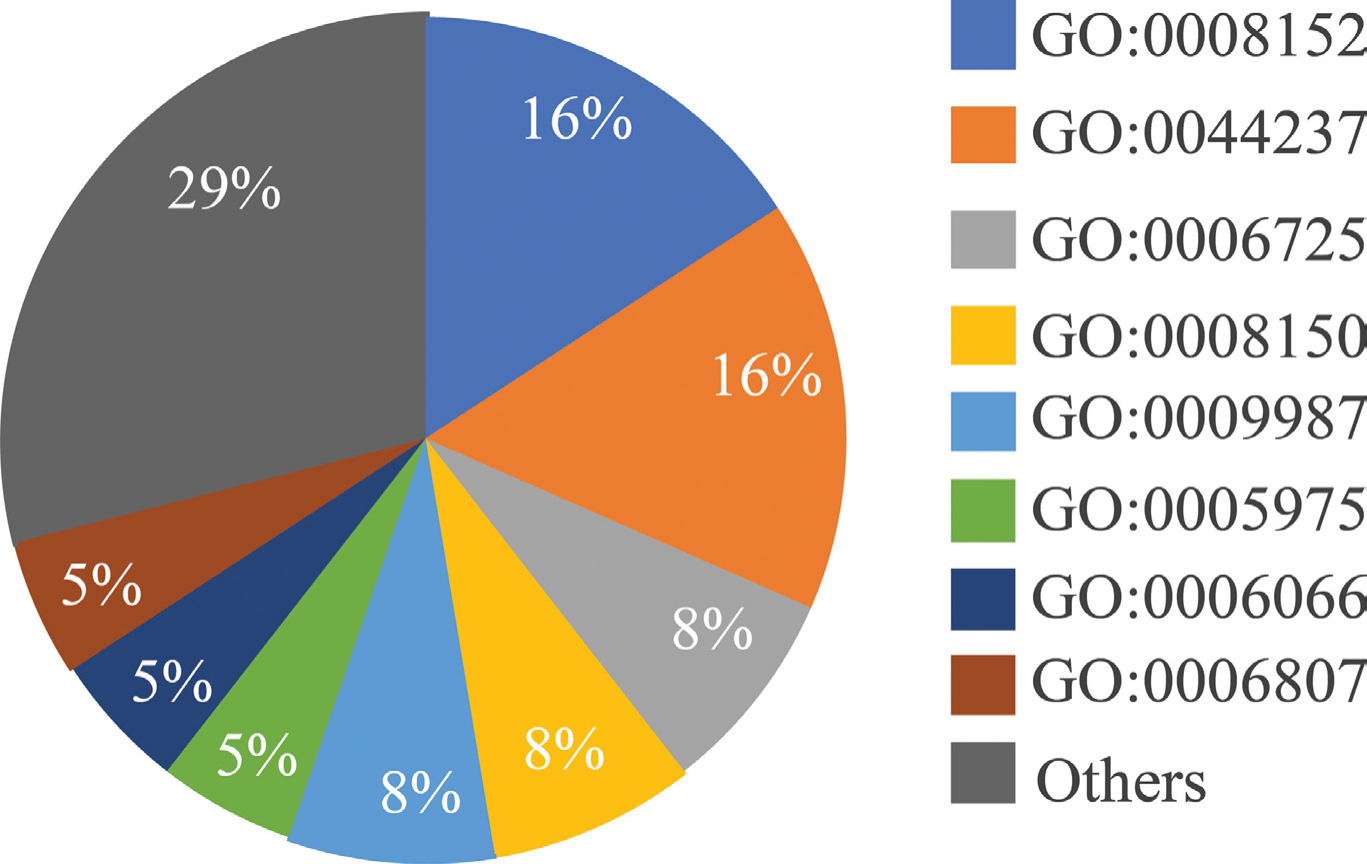
Percentage of genes amongst the protein clusters unique to EX14915 that are associated with biological process GO terms.

**Fig. 7.**
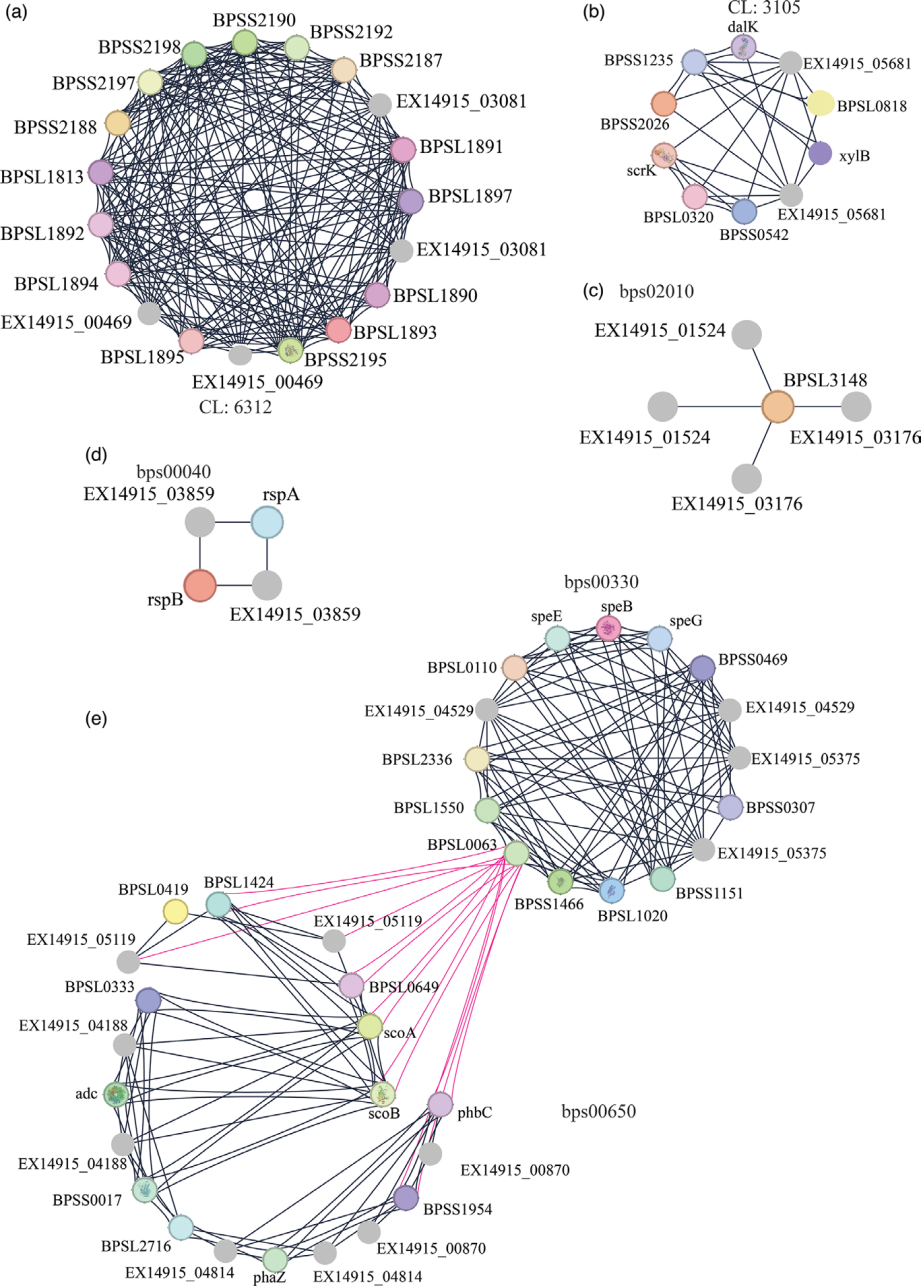
Protein interactions amongst the major clusters identified unique to EX14915. Of the 27 proteins identified, 24 were mapped to proteins in the database. For better visualization, nodes with no interactions were removed. The edges indicate a confidence score (>700) of interactions, and each cluster is identified as follows: (a) type II secretion system (CL: 6312), (b) pentose phosphate system (CL: 3105), (c) ABC transporters (bp02010), (d) pentose and glucuronate interconversions (KEGG: bps00040) and (e) two major clusters involved in arginine and proline metabolism (KEGG: bps00330) and butanoate metabolism (KEGG: bps00650) with shared interactions coloured in pink. Cluster images were created using Cytoscape v3.10.3 and using the Y-files circular layout plugin.

From the total proteins identified in both isolates as annotated by Prokka, 5,449 protein clusters were shared amongst the two isolates (Fig. 5a). The corresponding GO enrichments were shown in Fig. 5(b–d). Thirteen clusters were unique to the isolate from the first episode. From these 13 clusters, one functional association (oxido-reductase activity; GO: 0016491) was identified, and six clusters belonged to the cellular metabolic processes (GO: 0044237 and GO: 0008152) (Fig. 6). None of the eight clusters in EX7035 were associated with any GO terms. Using STRING v12.0 and searching against *Burkholderia* protein interaction networks, six protein network clusters were identified among the 13 unique clusters in EX14915 (Fig. 7). The cluster with the highest number of interactions is associated with butanoate metabolism (KEGG: bps00650) (Fig. 7e), whilst the next largest network had proteins associated with the type II secretion system (STRING cluster id CL-6312) (Fig. 7a).

### Genomic variants

A total of two SNPs were detected in the EX7035 isolate when EX14915 was considered as the reference sequence. Amongst these two variants, one (P1339T) was associated with the protein TcdB (locus tag: IOODPMLK_03814) that was misannotated as a hypothetical protein. The other variant was identified in the intergenic region between protein IOODPMLK_05780 and the end of its contig.

### Structural variants

Comparative analysis by alignment of genomes with the MSHR7901 genome highlighted greater fragmentation/non-identical regions in the EX7035 when compared with EX14915 (Fig. 1). A few regions from the EX14915 were deleted (had zero coverage) in the EX7035 genome as evidenced from the TIDDIT coverage BED file (Supplementary Material 1). In most of these regions, the mapping quality was below 60, and most of these regions had no coding sequences or were identified as hypothetical proteins (Table 2). Amongst the deletions within the contig 3, two copies putatively identified as cdiA2 (alleles 1 and 2) were within the span of the deletions. However, an additional allele (cdiA2_3) was detected. A simple blast search of these three alleles identified them as haemagglutinin repeat-containing proteins, indicating a possible mis-annotation by Prokka.

**Table 2.** Genomic regions of EX14915 with zero coverage in the isolate EX7035. In most of the regions, the mapping quality is <60. Only those contiguous regions spanning at least 100 bp are shown

Contig	Start	End	Span	Gene (location in ref)	Best hit	Function
3	14501	18001	3500	cdiA2 (allele 1: 13077–22505)	WP_207874369	Haemagglutinin repeat-containing protein
3	46001	49501	3500	cdiA2 (allele 2: 44456–53779)	WP_207865767.1	Haemagglutinin repeat-containing protein
333	1	2265	2264	IOODPMLK_05971 (486–1853)	No similarity	–
402	1	693	692	–	No similarity	–
156	15501	15955	454	IOODPMLK_04943 (15019–15891)	MDV2232457.1	Hypothetical protein
189	11001	11241	240	–	TOZ34005.1 (78% coverage)	Hypothetical protein
286	4001	4149	148	–	CAJ9830665.1 (44% coverage)	Glycosyl hydrolase family protein
18	62501	62607	106	–	–	–
86	29001	29103	102	–	–	–

## Discussion

Recurrent melioidosis due to relapse or reinfection has been reported from different parts of the world [15,16]. Differentiation between relapse and reinfection is vital for the epidemiology and management of melioidosis [17]. Following the initial presentation, recurrent melioidosis occurs within the first year in ~6% of patients. Relapse is more likely to occur in patients with multifocal and bacteremic melioidosis or when the duration of oral antimicrobial treatment for the first episode of infection was shorter than recommended [18]. The organism is capable of remaining latent in the host for several years, although the site of latency and its mechanism are not clear [19]. However, *B. pseudomallei* has been found within the nucleus of the host cells, indicating the potential site for the later recurrent episodes of melioidosis [20]. The choice and duration of oral antimicrobial therapy may also contribute to relapse [21]. However, relapse is well documented after successful treatment. Furthermore, mortality due to relapse is similar to that of the initial episode of melioidosis [22].

The recent advances in WGS have enabled it to be one of the most widespread molecular techniques used for epidemiological and evolutionary analysis of highly recombinogenic *B. pseudomallei* [1,2]. WGS is more robust compared to MLST by providing high resolution of closely related strains and can characterize their origin with greater accuracy [23]. Moreover, WGS provides a better understanding of transmission, virulence and antibiotic resistance along with the base-pair-level resolution of strain-level variations across the genome [24,25].

From the pair of clinical isolates collected, initial analysis by MLST revealed that both isolates were of the same ST (ST734). This ST was not detected amongst the five pairs of isolates from melioidosis relapse in a study from Thailand [5]. In a study from India, ST734 was identified in one of the blood isolates. Genetically, this ST can be related to multiple other STs, as it had multiple single (23), double (19) and triple (12) locus variants and 22 satellite STs. Due to its widespread similarity with other STs, it can be presumed to be a founder for many strains. Moreover, ST734 was known as an endemic clone in Australia, which was reported between 1999 and 2018 from human and environmental sources [26]. However, ST734 was rare or not identified from any other states in southern India, suggesting that there is a limited distribution of ST734 to southeastern India [27,30]. Two other STs (ST51 and ST56) were found to be persistent strains that caused infection across Asia [31].

The antimicrobial resistance genes were similar amongst the two strains, indicating that the relapse is not mediated by drug resistance. In the present study, both isolates carried blaOXA-57 and PenI but were susceptible to imipenem and meropenem phenotypically. Whilst this is an interesting find, similar mechanisms, especially in ST734, were reported previously. In a study by Amladi *et al*., ST734 was isolated from a patient with septic melioidosis and harboured the blaOXA-57 but was phenotypically susceptible [26]. Similarly, >90% of the 85 isolates with blaOXA-57 were phenotypically susceptible to imipenem [32].

The lipopolysaccharide (LPS) *in B. pseudomallei* is identified to be of three different genotypes – A, B and C. The LPS-A genotype is the most dominant genotype, with 97% of the isolates belonging to this class. This genotype is known to be associated with the first episode of melioidosis [33]. In the present study, both the isolates were identified as LPS-A genotypes using PCR described previously [4]. Previous studies identified varying levels of correlation between LPS genotype and relapse. In a study by Anuntagool *et al*., 27% of the 40 patients with relapse had differences in LPS genotype amongst the isolates, whilst in another study, no such correlation between LPS genotype and relapse was identified [33,34]. From our centre, we identified that the majority of the clinical isolates (64.7% of the 60 isolates) were of the LPS-A genotype [4].

For a direct comparison of the two isolates, the strain Ex14915 was chosen as the reference, as it is the isolate in the first episode of melioidosis. Initial QC of the assemblies revealed EX7035 to have multiple contigs, fragmented assemblies and an extremely low N50 (7,360 when compared with 34,519 in EX14915) (Table 1). One reason could be the lower number of reads in EX7035 (~2.08 million) when compared with EX14915 (~3.03 million) (Supplementary Material 2). To understand the effect of this variation, we used genome blast using BRIG to visualize the differences in genome assemblies. As expected, the isolate from the first episode had higher identities across the genome, whilst multiple breaks within the EX7035 genome were observed (Fig. 1). For this comparative visualization, the strain MSHR7901 was chosen as the reference, as it was of similar ST and had a low Mash distance when compared with both the isolates. We initially thus assumed a lower number of genes and functional regions within the EX7035 genome.

Although we had lower read counts in the relapse isolate EX7035, rather than attributing them to sequencing errors, we wanted to understand whether these represent true deletions. Moreover, after the reads were trimmed, the expected genome coverage was 81X when using 7.5 Mb as the genome size (estimated using seqtk in the Shovill pipeline) (Supplementary Material 2). One of the reasons why we attempted to identify SVs is that large deletions may occur in the genomes in the isolates from recurrent melioidosis, and can vary up to 22–800 kb deletions [5]. These deletions typically are within the intergenic regions, as is observed in the present study, and may be neutrally selective for evolution. Chromosome 1 is more frequently involved in such large-scale deletions, but as the complete genomes were not assembled in the present study, we could not identify which chromosome is involved with the structural variants.

To identify the structural variants within EX7035, the BAM file from Snippy was used for SV-calling with TIDDIT pipeline [14]. This tool uses mate-pair or paired-end sequencing data to identify intra- and inter-chromosomal translocations and large indels. It uses both supplementary alignments and discordant pairs for calculating the coverage and subsequently identifying structural variants. Both “cov” and “sv” functions in TIDDIT are used in the present study to estimate the coverage and identify SVs respectively. By default, it uses the 99.9 percentile to estimate the insert size and calls variants if there are at least three supporting read pairs. Using the coverage function, we identified multiple regions within EX7035 that had zero coverage (Supplementary Material 1). Amongst these regions, two alleles of haemagglutinin repeat-containing protein (cdiA2) were absent, along with an LysM domain-containing hypothetical protein (Table 2).

The cdiA2 proteins are involved in hydrolysis of phosphodiester bonds in tRNA molecules (GO: 0004549). Searching in the UniProt database, this protein is a part of the contact-dependent growth inhibition system (UniProt ID: I1WVY3) and can help in inhibiting the growth of other bacteria, therefore providing a survival advantage [35]. The LysM proteins belong to the PF01476 family and are short protein domains of ~40 aa in length. These domains have been identified in multiple bacteria and are involved in cell wall degradation. The domain is expected to be involved in peptidoglycan binding and is identified in the *Burkholderia cepacia* complex injectisome (BCI), which is a type III secretion system (T3SS) [36]. Other shorter deletions (<100 bp) were also identified, but the proteins associated within these regions were not analysed further.

We then identified varying numbers of RNA elements - both tRNA (*N*=77) and ncRNA (*N*=49) were in lower numbers in the isolate EX7035. However, the overall number of genes detected was slightly higher in this isolate (Table 1). We presumed that the high fragmentation of its genome led to incorrect annotations as hypothetical proteins or missed proteins due to split at the contig ends. To identify the functional impact of this annotation, we used VFDB to annotate the virulence factors and the NCBI AMRFinderPlus database to detect the antibiotic resistance genes [9,10]. The number of virulence genes was lower than expected in EX7035, and many genes had no coverage when read mapping was performed (Fig. 4). Amongst the genes that were unique to EX14915, functional annotation using the STRING database identified that many of these belong to the T3SS or flagellar assembly (Fig. 3). However, we caution the interpretation as most virulence genes had lower coverage within them and therefore led to missed annotation due to a lower number of reads in this genome.

To fully characterize the differences in proteomes, we then used the protein clustering tool OrthoVenn. Using an inflation value of 1.5, we identified that 5,449 protein clusters were common amongst both the isolates, and 13 unique clusters were identified in EX14915. Using the STRING database search, these unique clusters were annotated as involved with cellular metabolism (GO:0008152), like aromatic compound catabolism (GO:0006725), etc (Fig. 6).

To further characterize the genomic variants, using Snippy, the variants were predicted by mapping the reads, and those positions with a minimum base quality of 20 were chosen for further analysis. We identified very few differences between the strains. Only two mutations were detected, and one of the mutations was a substitution (P1339T) within a hypothetical protein (IOODPMLK_03814). Protein similarity searches identified this as a toxin TcdB middle/N-terminal domain-containing protein (WP_207865813) with high similarity (99.9%) and coverage (100%). This protein is mapped with STRING id BPSL0590 and is a putative membrane protein. This protein is known to irreversibly inactivate GTPases of the Rho family in *Clostridium difficile*, and macrophage rounding and decreased total cellular F-actin content in *B. cenocepacia* [37,38]. The substitution refers to a possible mutation in EX14915, as the TcdB WT protein has threonine at this site. However, this mutation does not form a part of the active site and hence may have no impact on the overall protein function [37].

Similar to our study, amongst the paired comparison of relapse isolates, very few differences were detected. The estimated within-host mutation rate was low for the *Burkholderia* genus and is on average 1–3.6 SNPs per genome per year [6]. In a study, only 16 mutations were identified amongst the six clinical isolate pairs with relapse [6], with a predominance of non-synonymous substitutions. Yet in another study, 0–5 SNPs were detected amongst paired isolates from two different patients [5]. One possible explanation for such low mutation rates was that purifying selection on synonymous mutations may limit their frequency. We identified only two variants, and both were transversions (A<->C). These transversions are assumed to occur due to oxidative damage to bacterial DNA and may occur either at higher (for C>A) or at lower frequencies (for A>C) [6].

Although we were able to identify the genetic variants, our study did not employ hybrid genome assembly strategies due to limited financial constraints. Using a hybrid genome assembly may have substantially validated our results by achieving near-complete genome assemblies. Nevertheless, our study merits employing various bioinformatic methods to establish the role of genomic variants on possible bacterial physiology and disease relapse. Specifically, we were able to establish that the second episode was a relapse and not a reinfection with a different strain. Our findings closely match those previously found amongst such clinical pairs with low SNP and higher structural variants.

We also identified a clinical-environmental pair of isolates from another patient and would like to understand the genetic differences between them. Such comparative genomic studies are rarely conducted in India, and perhaps ours was the first study to report such findings in this neglected pathogen. It becomes necessary thus to continuously monitor the genomes of the pathogen to understand the strain-specific within-host evolution.

## Conclusions

Based on genome sequence analysis, we identified that both isolates shared similar proteome and virulence genes and indicated that the relapse may have possibly happened due to host-related factors rather than large-scale strain variations. There was no difference in the antimicrobial resistance pattern or the genes involved in drug resistance, indicating that therapy during the initial episode was largely successful and that the relapse was not due to a persistor subpopulation.

## Supplementary material

10.1099/jmm.0.002003 Supplementary Material 1.

10.1099/jmm.0.002003 Supplementary Material 2.

## Abbreviations

LPS: lipopolysaccharide
WGS: whole-genome sequencing
