# Supplementary material for: Comparative genomic analysis of paired clinical isolates from a patient with recurrent melioidosis reveals a low within-host mutation rate
Source: J Med Microbiol. 2025 Apr 15;74(4):002003. doi: 10.1099/jmm.0.002003 (PMC12282283; doi:10.1099/jmm.0.002003)
Supplement: Supplementary Material 2. [file jmm-74-02003-s002.pdf]

Metric	Ex14915 (1st episode)	Ex7035 (2nd episode)
Read 1 - Total reads (pre-filter)	3024832	2075526
Read 1 - Total bases (pre-filter)	451243639	303857010
Read 2 - Total reads (pre-filter)	3024832	2075526
Read 2 - Total bases (pre-filter)	448956449	307330481
Read 1 - Total reads (filtered)	2774848	1806709
Read 1 - Total bases (filtered)	330508700	215887597
Read 2 - Total reads (filtered)	2774848	1806709
Read 2 - Total bases (filtered)	328217423	216493983
Q20 ratio (filtered/raw) – read 1	0.7321	0.71267
Q20 ratio (filtered/raw) – read 2	0.73136	0.706378
Average coverage*	86.3149	66.3103
Number of mutations*&	1236	1112

Supplementary table 1: Raw data QC. *Estimated using GCA_017378195.1 as the reference genome

&Includes both synonymous and non-synonymous SNPs identified by the Snippy pipeline

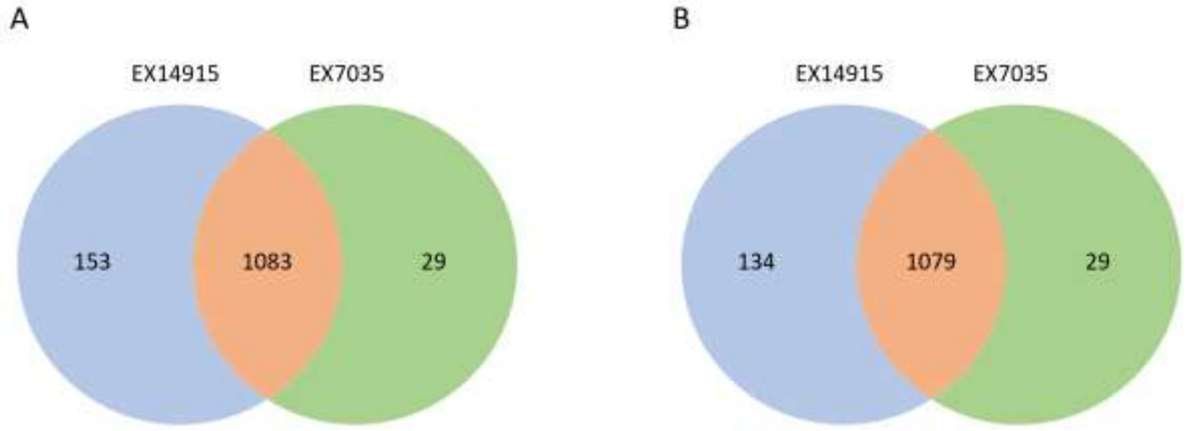

Supplementary figure: Mutations (SNPs) shared and unique to the isolates among the two episodes before (A) and after (B) down-sampling to match genome expected coverages. There was less than 0.9-fold (134/153) decrease in number of SNPs unique to EX14915 after down-sampling. Down-sampling of EX14915 was performed at a proportion 0.76 after comparing the average coverages of EX14915 and EX7035 w.r.t. GCA_017378195.1. Subsequently Snippy pipeline with previously mentioned arguments was used for variant calling for EX14915 using the down-sampled reads.
